# Ultrasound evaluation of a new surface reference line to describe sural nerve location and safe zones to consider in posterior leg approaches

**DOI:** 10.1007/s00167-022-07294-8

**Published:** 2022-12-26

**Authors:** Pablo Ruiz-Riquelme, Daniel Poggio-Cano, Xavier Sala-Blanch, Daniel Cuéllar Bernal, Albert Baduell, Rubén Garcia-Elvira, Enrique Adrián Testa

**Affiliations:** 1 Master Fellow Foot and Ankle Surgery, Universitat de Barcelona, Barcelona, Spain; 2 Department of Orthopedic and Traumatology, Hospital Clínico La Florida / Clínica Las Condes, Santiago, Chile; 3 School of Medicine, Finis Terrae University, Santiago, Chile; 4 Foot and Ankle Surgery Unit, Department of Orthopedics and Traumatology, Hospital Clinic de Barcelona, Universitat de Barcelona, Barcelona, Spain; 5 Anesthesiology, Department of Anesthesiology, Hospital Clinic de Barcelona, Universitat de Barcelona, Barcelona, Spain; 6 Anatomy and Human Embriology Unit, School of Medicine, Universitat de Barcelona, Barcelona, Spain; 7 Clínica Santa Ana – Clínica Norte, Cucuta, Colombia; 8 Department of Orthopedic and Traumatology, Ospedale Regionale di Bellinzona e Valli, Bellinzona, Switzerland

**Keywords:** Sural nerve, Ultrasonography, Peripheral nerve injuries, Leg injuries, Posterior leg and ankle approaches

## Abstract

**Purpose:**

Several authors have described methods to predict the sural nerve pathway with non-proportional numerical distances, but none have proposed a person-proportional, reproducible method with anatomical references. The aim of this research is to describe ultrasonographically the distance and crossing zone between a surface reference line and the position of the sural nerve.

**Methods:**

Descriptive cross-sectional study, performed between January and April 2022 in patients requiring foot surgery who met inclusion criteria. The sural nerve course in the posterior leg was located and marked using ultrasound. Landmarks were drawn with a straight line from the medial femoral condyle to the tip of the fibula. Four equal zones were established in the leg by subdividing the distal half of the line. This way, areas based on simple anatomical proportions for each patient were studied. The distance between the marking and the ultrasound nerve position was measured in these 4 zones, creating intersection points and safety areas. Location and distances from the sural nerve to the proposed landmarks were assessed.

**Results:**

One-hundred and four lower limbs, 52 left and 52 right, assessed in 52 patients were included. The shortest median distance of the nerve passage was 2.9 mm from Point 2. The sural nerve intersection was 60/104 (57.7%) in Zone B, 21/104 (20.1%) in Zone C and 19/104 (18.3%) in Zone A. Safety zones were established. Average 80.5% of coincidence in sural nerve localization was found in the distal half of the leg, in relation to the surface reference line when comparing both legs of each patient.

**Conclusions:**

This study proposes a simple, reproducible, non-invasive and, for the first time, person-proportional method, that describes the distance and location of the main areas of intersection of the sural nerve with points and zones (risk and safe zones) determined by a line guided by superficial anatomical landmarks. Its application when surgeons plan and perform posterior leg approaches will help to avoid iatrogenic nerve injuries.

**Level of evidence:**

IV.

## Introduction

Sural nerve (SN) injury may cause hypoesthesia, neuropathic pain, and even affect the patient's psychological state and quality of life [3, 4, 26, 37]. Studies about the SN pathway have described different morphological patterns in its proximal formation [35, 38, 39, 43]. The most frequent pattern runs as a single nerve between 83.7 [38] to 97% [39] in the distal 1/2 or 1/3 of the leg, respectively. Some posterior leg approaches have a high risk of SN injury [30, 47]. These approaches are often used in acute [12, 35, 47] or chronic [16, 44] Achilles pathology, ankle arthroscopy [2, 23, 34] or ankle fractures [18, 29, 33]. Several authors have attempted to predict the SN location with distance units from the Achilles tendon [6, 10, 14, 20, 46] or the lateral malleolus [9, 14, 15, 18, 24, 30], but these units can be altered according to height [15, 20], leg length [6] or body mass index (BMI) [15]. Furthermore, no authors have proposed a reproducible method that is unaffected by these factors (i.e., based on personal proportions).

Knowledge of SN anatomy, preoperative planning, proper surgical technique and identification of safety zones, can help to avoid iatrogenic injuries [3, 40, 42]. The location of the SN and safe zones for surgical incision has been evaluated in our center by tracing a line from the medial femoral condyle to the lateral malleolus with apparently good results. It was hypothesized that the same trajectory will be accurately observed using ultrasound (US) in the SN and the reference line in the distal leg [5, 20, 21, 28, 35, 45]. The aim of this research is to describe ultrasonographically the distance and crossing zone between the surface reference line and the position of the SN.

## Material and methods

This cross-sectional analytical observational study has the approval of the ethics committee of the Hospital Clinic Barcelona (HCB/2022/0228) and all participants provided informed consent. Patients over 18 years of age who underwent ambulatory non-traumatic foot surgery with regional anesthesia between January and April 2022, with normal neurovascular and soft tissues of the leg were included. Patients with previous surgeries, wounds, or scars in the posterior area of the leg and those with a history of peripheral neuropathy were exclued. A demographic characterization of the enrolled patients was carried out, considering age, gender, lower extremity length, weight, height, BMI and laterality of the lower extremity. For every patient, the distal half of the two legs was analyzed, considering the SN runs as a single nerve [38, 39] and evaluating five points that generate 4 zones of equal size. This way, we studied reproducible areas based on simple anatomical proportions for each patient. Reference points, laterality and the crossing zone of the SN in relation to the surface reference line were described, as well as safe areas, such as those in which the SN does not intersect and has a path that moves away from the reference line. Analysis of the concordance of the distances to points 1, 2, 3 and 4 of the SN, between the left and right leg, and differences between both legs were evaluated.

### Assessment of the sural nerve path

Prone position, keeping the feet off the edge of the examination table to avoid plantar flexion of the ankle. To facilitate localization of proximal anatomical landmarks, the knees were semi flexed (30 degrees approximately) before resting the leg on the examination table.

**Fig. 1:**
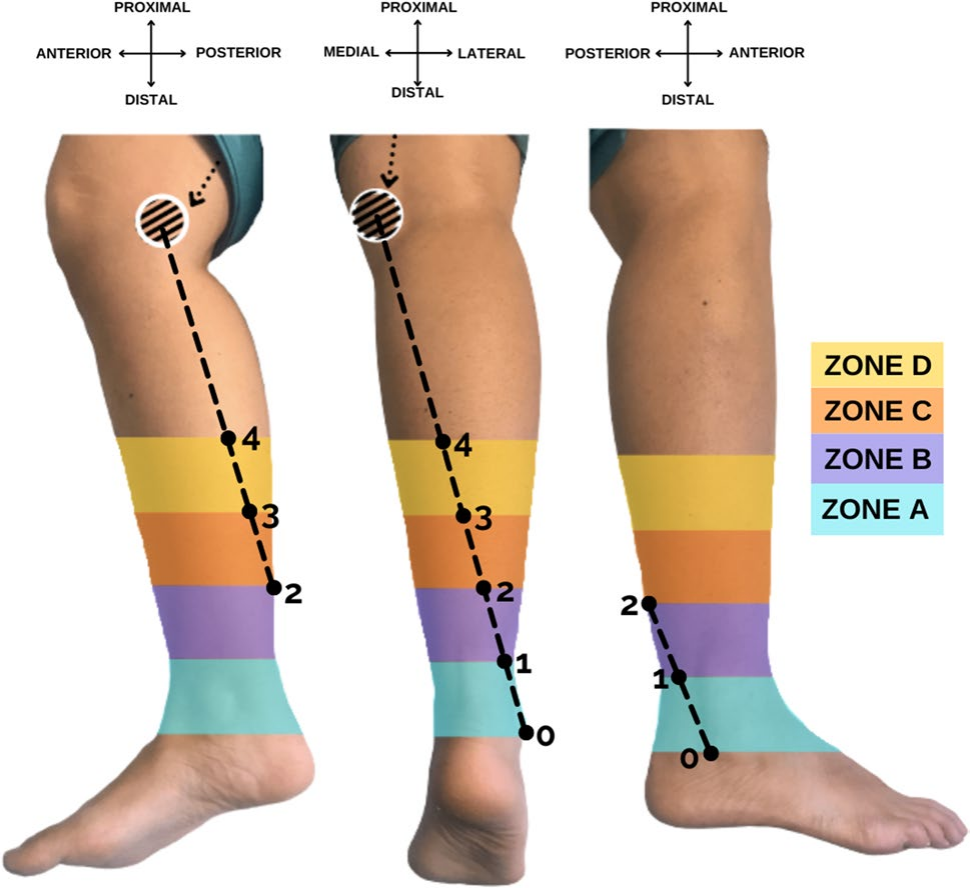
Description of the surface reference line, reference points and zones on a right leg. From left to right, medial, posterior, and lateral views of the leg. Striped circle: medial femoral condyle; Dotted arrow: hamstring tendon; Dotted line: surface reference line; Point 0: fibular tip. Subdivision of the distal half of the leg into five points and four zones listed from distal to proximal

For the superficial anatomical assessment, a straight line was drawn by the principal author between two anatomical landmarks. The line went from a point between the medial aspect of the medial condyle and the postero-lateral aspect of the medial hamstring tendon to the most distal and posterior aspect of the lateral malleolus (Fig. 1). A straight line was drawn using a 2 mm rope as a guide to join the proximal and distal anatomical references. The line was measured and divided into two segments of equal length, the distal segment was further divided into four sections which were identified by points 0, 1, 2, 3 and 4 (distal to proximal). These points subsequently delimited four equal quarters of the distal half of the leg, which were classified as zones A, B, C and D (distal to proximal) (Fig. 1).

US evaluation was performed by an operator with more than 20 years of experience in ultrasound-guided regional anesthesia, using 5 to 13 Hz linear transducer (CANON APLIO I800 18L), musculoskeletal filter, focus 0.75, nerve depth with average image 2 cm. The cross-sectional area of the posterior aspect of the distal half of the leg was evaluated, using the posterior calcaneal tuberosity as the distal limit. The SN was identified along its distal to the proximal course. When not identified at one point, the operator moved on to the proximal point, then followed its course distally. When it was difficult to see the nerve, the lesser saphenous vein was used as a close reference [17]. The position of the reference line was verified by two certified orthopedists.

**Fig. 2:**
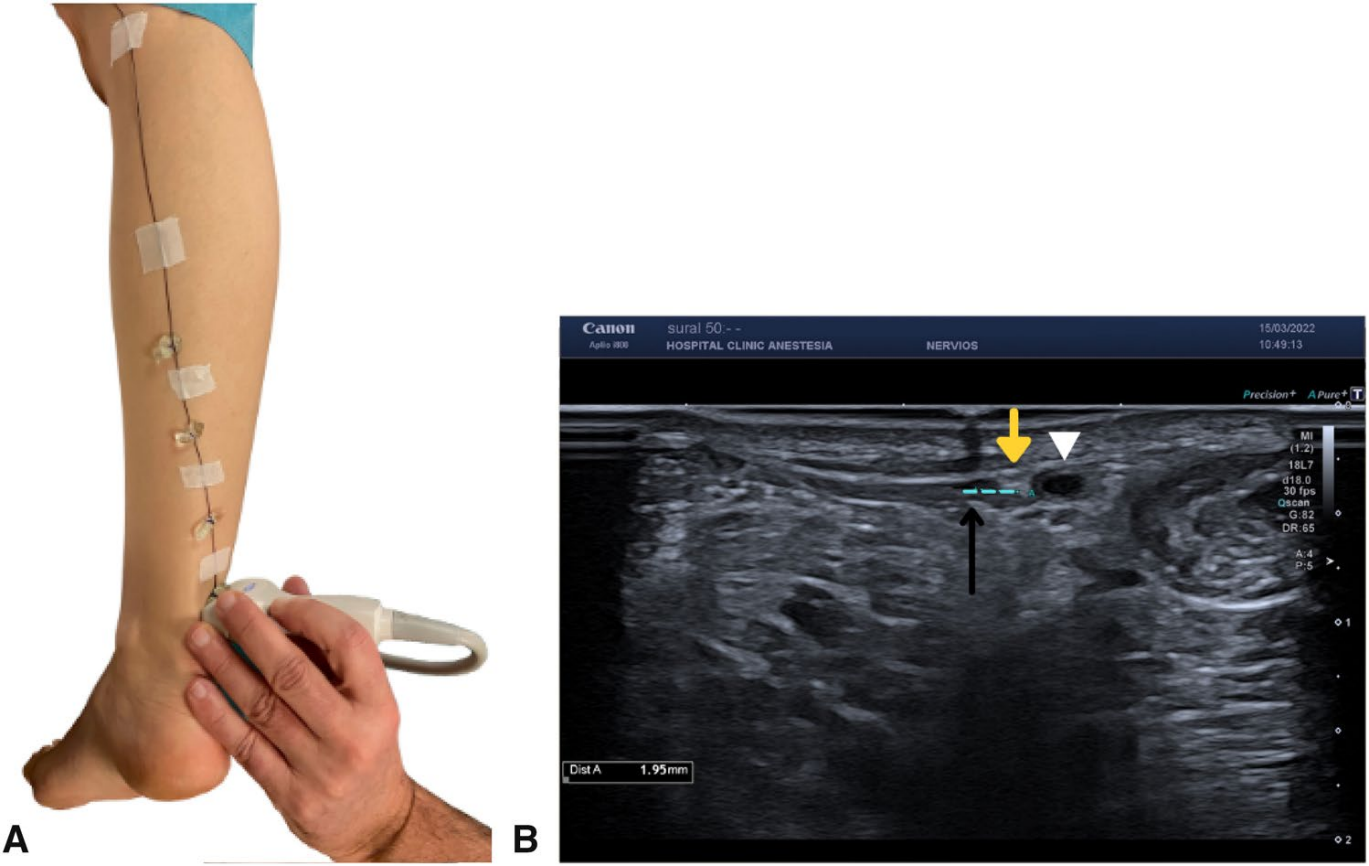
Sural nerve ultrasound evaluation and its relationship with surface reference line on a left leg. **A** Clinical position of the sonography for the evaluation of the sural nerve in relation to Point 1. **B** Sonographic image shows the distance (dotted light blue line) between the sural nerve (yellow arrowhead) and the acoustic shadow of the line (black arrow). White triangle: saphenous vein

An evaluation of the concordance between the SN pathway and the surface reference line was performed by measuring the distance between the SN and the described points of the line in the medial–lateral axis. The acoustic shadow of the line and the position of the SN were measured ultrasonographically in millimeters (Fig. 2). In addition, the intersection of the SN with the surface reference line was assessed.

The percentage of concordance of the SN pathway was evaluated by comparing the two lower extremities of the same individual. Laterality (medial–lateral) and the crossing zone between the SN, the four points and four zones were evaluated, respectively.

### Statical analysis

A sample size calculation was postulated assuming an SN concordance between the two legs greater than 15% of that reported in the literature [38, 39], with an alpha error of 5% and a power of 80%. A sample size of 96 lower limbs (48 patients) was determined.

Continuous variables (age, weight, height, BMI, length of surface line, distance between the nerve and the surface line points) had a nonparametric distribution, so median and range was used. The position of the SN, in relation to the reference points, distance (lateral-medial) and reference zones of both legs were described with medians and range as well and categorical variables with frequency and percentage. Differences in the median distance from the reference points (1, 2, 3 and 4) of the surface reference line to the passage of the SN were evaluated with. Wilcoxon rank tests. A Fischer test was used to compare the SN crossing zone with the reference line between both legs (Table 1). Finally, the percentage of concordance of the location and crossing zone of the SN regarding the surface reference line between both legs was described (Table 2).

**Table 1:** Sural nerve crossing zones of the reference line in all sample and between both legs

Crossing zones %(*n*)	Total sample*** (*n* = 104)	Right leg** (*n* = 52)	Left leg (*n* = 52)	*P* value*
Zone A	18.3% (19)	19.2% (10)	17.3% (9)	n.s
Zone B	57.7% (60)	61.5% (32)	53.8% (28)	n.s
Zone C	20.2% (21)	19.2% (10)	21.2% (11)	n.s
Zone D	1.9% (2)	0% (0)	3.8% (2)	n.s

*n.s.: non-significant differences between both legs

**In two right legs the sural nerve coincided with the reference line in

Two zones simultaneously

***In two right and three left legs the sural nerve did not cross the reference line in any zone

**Table 2:** Sural nerve crossing zone concordance with the reference line when comparing both legs per person

References zones	Concordance	
	*n*º	%
Zone A	0/52	0
Zone B	19/52	36
Zone C	5/52	10
Zone D	0/52	0
Total concordance	24/52	46

Data analysis was performed with STATA 14 Texas corp. ind.

## Results

One-hundred and four lower limbs, 52 left and 52 right, assessed in 52 patients were included. Due to technical feasibility, it was not possible to collect a sufficient sample to obtain significant results when comparing both limbs (subgroup analysis), but according to the literature enough sample [1, 6, 21, 35] to evaluate the trajectory and morphology of SN.

52% of the participants were male (54/104 limbs) and 48% female (50/104 limbs), median age was 45 (22–74). Median height was 164 (150–180) cm with a median BMI 23.3 (17.3–39.7) kg/cm^2^. The surface reference line had a median length of 44 (36–48) cm. The medial–lateral location of the SN in relation to the traced points of the line was described according to a percentage (Table 3) and median distance (Table 4). In 95.2% of the cases, SN intersected the surface reference mainly in Zone B (Table 1). In the rest 5/104 members (4.8%), the SN passed laterally to the line. An intersection between SN and the reference line was found to occur in two zones simultaneously, Zones B and C, in 2/104 limbs (1.9%).

**Table 3:** Percentage of sural nerve medial–lateral location respect to the traced points of the reference line

	References points	Sural nerve localization %(*n*)		
		Medial	On the point	Lateral
Total Sample (*n* = 104)	Point 1	91.4% (95)	3.8% (4)	4.8% (5)
	Point 2	28.8% (30)	10.6% (11)	60.6% (63)
	Point 3	2.9% (3)	7.7% (8)	89.4% (93)
	Point 4	0% (0)	0% (0)	100% (104)
Female (*n* = 50)	Point 1	96% (48)	0% (0)	4% (2)
	Point 2	30% (15)	12% (6)	58% (29)
	Point 3	0% (0)	8% (4)	92% (46)
	Point 4	0% (0)	0% (0)	94% (50)
Male (*n* = 54)	Point 1	87% (47)	7.4% (4)	5.6% (3)
	Point 2	27.8% (15)	9.2% (5)	63% (34)
	Point 3	5.6% (3)	7.4% (4)	87% (47)
	Point 4	0% (0)	0% (0)	100% (54)

**Table 4:** Distance of sural nerve medial–lateral location respect to the traced points of the reference line

References points	Sural nerve median distance (mm)			
	Medial	Range	Lateral	Range
Point 1	6.0	(1.0–22.0)	5.0	(1.9–8.5)
Point 2	2.9	(1.0–8.8)	4.0	(1.0–13.3)
Point 3	3.0	(1.0–10.0)	11.2	(1.0–29.0)
Point 4	–	–	22.1	(3.9–50.0)

When comparing the SN in relation to the surface reference line in the two extremities of the same patient, A mean of 80.5% SN location concordance between the four zones. SN pathway had a 46.2% of coincidence in the crossing zones (Table 2) with non-significant differences in these crossing zones (Table 1). SN location in relation to the reference points, 83% passed medially in Point 1, 56% passed in Point 2 (7 medially and 22 laterally), 83% did so in Point 3 (42 laterally, 1 at the same point) and 100% passed laterally through Point 4.

**Fig. 3:**
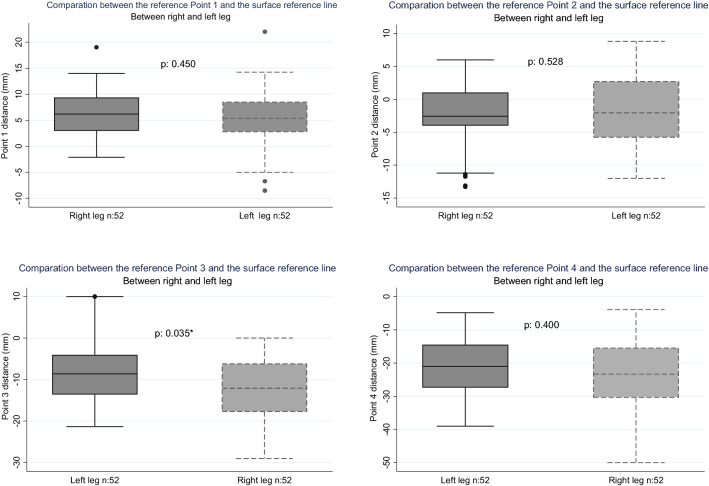
Sural nerve distribution in patients (*n *= 104) and its relation to the surface reference line. **A** Location and distance of the sural nerve in relation to the reference points. On the vertical-axis positive values represent a medial location and negative values a lateral location. **B** Representation of the distribution of the sural nerve in relation to the surface reference line and its points on a right leg

There were non-significant differences regarding the SN distance in relation to the reference points, except for Point 3 *(p* = *0.035)* (Fig. 3). Upon comparison of the SN distance with the reference points, there were non-significant differences in the dichotomous evaluation of the variables of age, BMI, height and sex (Table 5).

**Table 5:** Analysis between dichotomously variables, both legs, and distance from the sural nerve to reference points

Variable	Outcomes p50 (range)	Right leg	Left leg	*P* value*
Age < 40 years (*n* = 24)	Distance Point 1 (mm)	606	571	n.s
	Distance Point 2 (mm)	592	585	n.s
	Distance Point 3 (mm)	568	608	n.s
	Distance Point 4 (mm)	583	593	n.s
Age ≥ 40 years (*n* = 28)	Distance Point 1 (mm)	768	829	n.s
	Distance Point 2 (mm)	780	816	n.s
	Distance Point 3 (mm)	846	751	n.s
	Distance Point 4 (mm)	863	734	n.s
BMI < 25 kg/m2 (*n* = 34)	Distance Point 1 (mm)	1170	1176	n.s
	Distance Point 2 (mm)	1135	1212	n.s
	Distance Point 3 (mm)	1129	1217	n.s
	Distance Point 4 (mm)	1180	1167	n.s
BMI ≥ 25 kg/m2 (*n* = 18)	Distance Point 1 (mm)	343	324	n.s
	Distance Point 2 (mm)	349	318	n.s
	Distance Point 3 (mm)	377	290	n.s
	Distance Point 4 (mm)	370	297	n.s
Height < 170 cm (*n* = 24)	Distance Point 1 (mm)	590	586	n.s
	Distance Point 2 (mm)	550	626	n.s
	Distance Point 3 (mm)	571	606	n.s
	Distance Point 4 (mm)	584	593	n.s
Height < 170 cm (*n* = 28)	Distance Point 1 (mm)	799	798	n.s
	Distance Point 2 (mm)	830	767	n.s
	Distance Point 3 (mm)	850	746	n.s
	Distance Point 4 (mm)	859	737	n.s
Female (*n* = 25)	Distance Point 1 (mm)	551	724	n.s
	Distance Point 2 (mm)	524	751	n.s
	Distance Point 3 (mm)	504	771	n.s
	Distance Point 4 (mm)	611	665	n.s
Male (*n* = 27)	Distance Point 1 (mm)	796	689	n.s
	Distance Point 2 (mm)	729	757	n.s
	Distance Point 3 (mm)	727	759	n.s
	Distance Point 4 (mm)	789	696	n.s

*n.s.: non-significant differences

## Discussion

The most important finding of the present study was the existence of a common pathway between the SN and the surface reference line, both descending from the proximal-medial to the distal-lateral side of the leg. In addition, their crossing zones and the distances between them were objectified using US.

At least eight different patterns of SN have been described in the literature [35, 38, 39, 43] their variability being mainly located in their proximal formation. The most frequent pattern is formed by the union between the lateral and the medial sural cutaneous nerve (LSCN and MSCN) or the common peroneal nerve and MSCN and it is type 1, between 51.5 and 81% [21, 25, 38, 39, 43]. 83.7% of this pattern descends and forms a single SN in the distal half of the leg [30]. The nerve’s distal course was identified using US in all patients in our series. The symmetry of the SN between both legs of the same patient varies between 64.1 and 66.6% according to systematic reviews[38, 39]. In our series, the SN and the reference line were found to have a mean of 80.5% coincidence in the studied zones, and about half of this number in the crossing zone location (Table 2), which favors predicting its path. A significant difference was only observed when comparing the SN distance at Point 3 between both legs (Fig. 3). In such a case, the median location of both was lateral to the point with a median distance of 12.4 mm and 8.6 mm for the left and right leg. This difference could be attributed to a few nerves with a medial passage in the right leg.

**Fig. 4:**
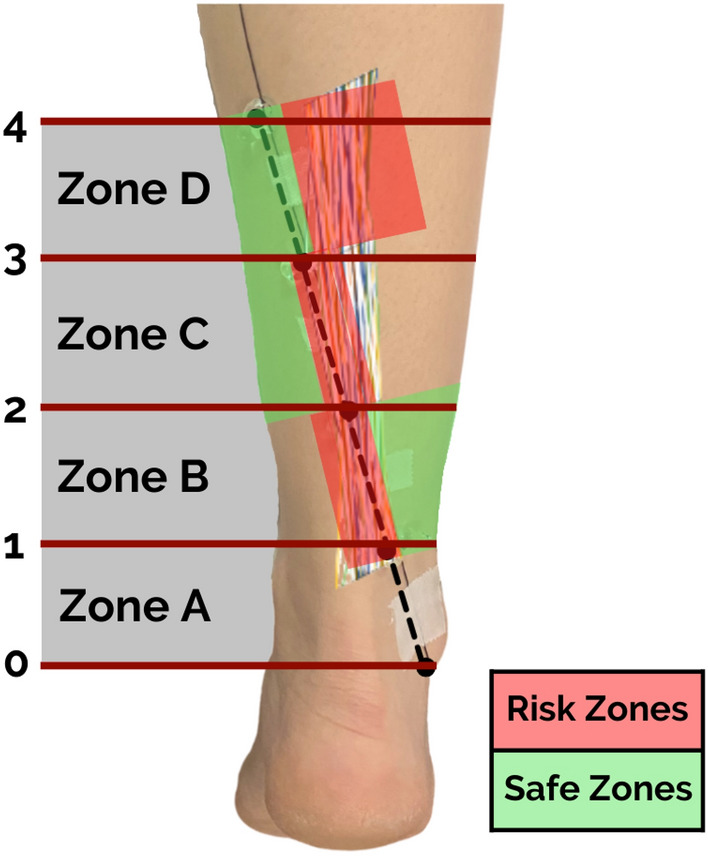
Prediction of safe and risk zones for sural nerve injury from the surface reference line. Risk Zones were defined like areas with a high probability of sural nerve injury during the approach, considering the points and zones of the reference line; it is recommended to identify the nerve in these risk zones. Safe zones were defined like areas with a low probability of nerve injury

The sural nerve has a risk of injury with posterior leg approaches. Minimally invasive surgery (MIS) of Achilles tendon rupture has a risk ranging between 5.5% and 60% [12, 47], up to a 27% with the Achillon technique [36]. This evidence discourages the extensive use of such techniques and has led to the search for new methods to reduce the risk. In percutaneous surgery, the risk has been reduced by exposing the SN through small incisions [22, 26] or through the identification of the trajectory of the SN using US mapping [11] or intraoperative US support [8, 32, 48]. In addition, open surgery has a lower risk of SN injury versus MIS [13, 27]. Other approaches such as the posterolateral ankle approach have a high risk of SN injury, from 66.3 to 100% according to clinical study using US [30] or magnetic resonance imaging (MRI) [15]. Some authors also mention that when using this approach, SN can be found in 83% of the cases reported [18]. A close path between the SN and the superficial reference line was observed in the distal half of the leg in our study. The SN crosses the line in 57.7% of Zone B, 20.2% of Zone C and 18.3% of Zone A. The nerve was found closest to the reference line in Zone B, at 2.9 mm medially and 4 mm laterally, followed by Zone A (Table 4). Therefore, according to our series, incisions medial to the reference line with a minimum distance of 20 mm, 10 mm, 10 mm and 0 mm from Points 1, 2, 3 and 4, respectively, would be safe to perform (Table 4). Proximal zones and points may be useful in revision surgery or chronic Achilles tendon rupture surgery [16, 31, 41, 44] or in some Achilles tendon lengthening techniques [7, 10, 19, 41]. The central zones may be useful in acute Achilles pathology surgeries [12, 22, 35, 47] and distal zones in arthroscopic surgeries [2, 23, 34] or ankle fractures [18, 29, 33]. This new reference line can be easily reproduced intraoperatively (using suture or electric scalpel wire) by the surgeon when planning the approach. Considering its path as similar to the SN would allow for predicting safe or at-risk areas for nerve injury (Fig. 4). In the latter a meticulous dissection and visualization of the nerve would be appropriate to prevent injuries.

Currently, methods to assess areas of increased risk for SN injury continue to be proposed. These methods propose distances in centimeters (metric unit) from anatomical landmarks (achilles or fibula) in patients, without considering their characteristics (non-proportional methods). The location of the SN lateral to the Achilles tendon, from its insertion site or from different areas of the tendon, has been described in cadaveric [14, 46] or clinical studies using US [20]. The crossing zone of the SN and the tendon has also been described as being approximately 8–10 cm from the insertion site of the Achilles tendon [6, 46]. Similarly, the medial location of the SN in relation to the fibula has been described [9, 14, 15, 18, 24, 30]. However, the problem with these metric length measurements is that they vary according to patient height [15, 20] leg length [6], BMI [15] or whether the Achilles tendon has ruptured [24]. The proposed surface reference line, made using 5 landmarks, is a useful tool to estimate the trajectory of the SN as it determines proportional zones of the leg for each patient (proportional method) to evaluate the SN and is unaffected by age, height, BMI and sex. To our knowledge, the existing studies propose anatomical references and absolute distances to predict the SN pathway and only a few propose reproducible methods without surgically exposing the nerve using US [8, 11, 32, 48]. In this new method, the relationship between the SN and the line was described using US. However, US is not required to employ it.

This study has some limitations. First, there are factors that may alter the measurements like the possible difficulties in locating the anatomical landmarks in obese patients, subjects with rotational or angular alterations of extremities (coronal or sagittal plane) or in patients with pathologies with soft tissue alterations, such as fractures or compartment syndrome. Secondly, the measurements are limited to only four points of the line. Third, the subgroup results must be confirmed by a bigger sample. Forth, the measurements performed in this study were performed using US, an operator-dependent test. Even though they were performed by an expert with more than 20 years of experience, the use of US cannot validate the method since a diagnostic study design is required. Another limitation of this study is that only cases with an intact Achilles tendon were chosen. MacMahon et al. [24] demonstrated that the nerve pathway changes in relation to the Achilles tendon when comparing patients with and without rupture. The surface reference line could be a valid alternative to the lateral edge of the Achilles tendon used in most studies as a reference, but further research is necessary to reduce bias and validate the method.

**Fig. 5:**
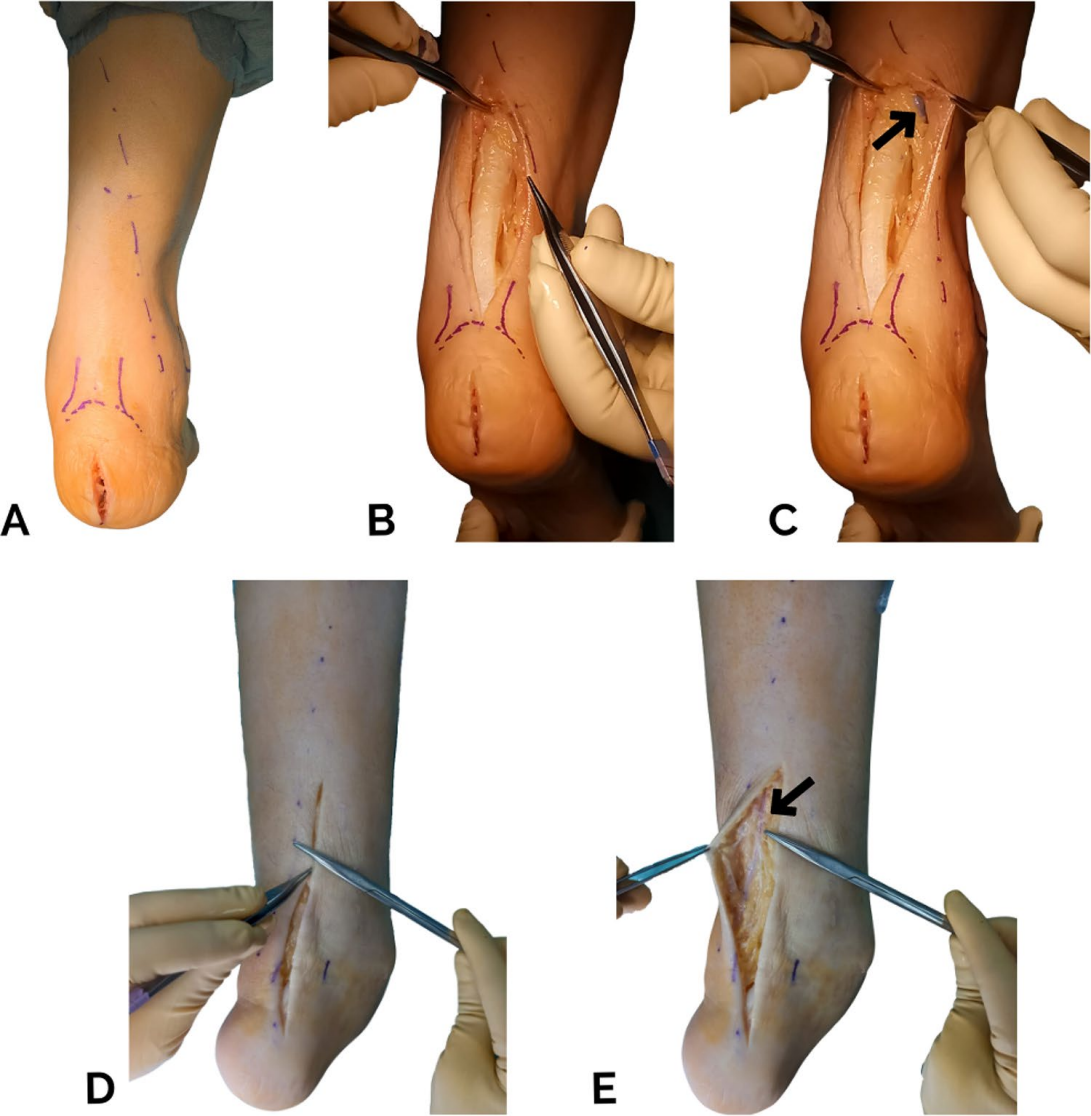
Clinical application of the new surface reference line during posterior leg approaches surgery to avoid a sural nerve lesion. Right posterior leg in the prone position, pre-incision with the surface reference line (**A**), central approach (**B**) and exposition of the saphenous vein and sural nerve (black arrow) near the line (**C**). Left posterior leg in the prone position, central approach and pointing the nerve location with the tip of the scissors (**D**) and exposition of the saphenous vein and sural nerve (black arrow) near the line (**E**)

The clinical application of this study is to provide an intraoperative tool to those surgeons who need to perform a posterior approach to the leg. The path of the SN (Fig. 5) and potential risk zones (Fig. 4) can be predicted using this tool based on a surface reference line, without the need for ultrasound. Looking for the nerve in such risk zones during surgery is recommended to avoid its injury.

## Conclusions

This study proposes a simple, reproducible, noninvasive and, for the first time, person-proportional method that describes the distance and location of the main areas of SN intersection with points and areas (risk and safe zones) determined by a line guided by superficial landmarks. Although new clinical studies with larger series considering different pathologies are needed to validate this method, we believe that incorporating this simple tool into surgical practice is a non-costly and simple way to plan and execute posterior leg and ankle approaches to minimize the iatrogenic nerve injury.
