# Correction to: Ultrasound evaluation of a new surface reference line to describe sural nerve location and safe zones to consider in posterior leg approaches

**DOI:** 10.1007/s00167-023-07323-0

**Published:** 2023-02-14

**Authors:** Pablo Ruiz-Riquelme, Daniel Poggio-Cano, Xavier Sala-Blanch, Daniel Cuéllar-Bernal, Albert Baduell, Rubén Garcia-Elvira, Enrique Adrián Testa

**Affiliations:** 1 Master Fellow Foot and Ankle Surgery, Universitat de Barcelona, Barcelona, Spain; 2 Department of Orthopedic and Traumatology, Hospital Clínico La Florida / Clínica Las Condes, Santiago, Chile; 3 School of Medicine, Finis Terrae University, Santiago, Chile; 4 Foot and Ankle Surgery Unit, Department of Orthopedics and Traumatology, Hospital Clinic de Barcelona, Universitat de Barcelona, Barcelona, Spain; 5 Anesthesiology, Department of Anesthesiology, Hospital Clinic de Barcelona, Universitat de Barcelona, Barcelona, Spain; 6 Anatomy and Human Embriology Unit, School of Medicine, Universitat de Barcelona, Barcelona, Spain; 7 Clínica Santa Ana – Clínica Norte, Cucuta, Colombia; 8 Department of Orthopedic and Traumatology, Ospedale Regionale di Bellinzona e Valli, Bellinzona, Switzerland

**Correction to: Knee Surgery, Sports Traumatology, Arthroscopy** 10.1007/s00167-022-07294-8

Original version of this article was published with incorrect version of figure 1 and error in author name. These errors now corrected.

1) Revised version of Fig. 1 updated.

2) Error in co-author name corrected as Daniel Cuéllar-Bernal (not as Daniel Cuéllar Berna).

The original article has been corrected.

**Fig. 1:**
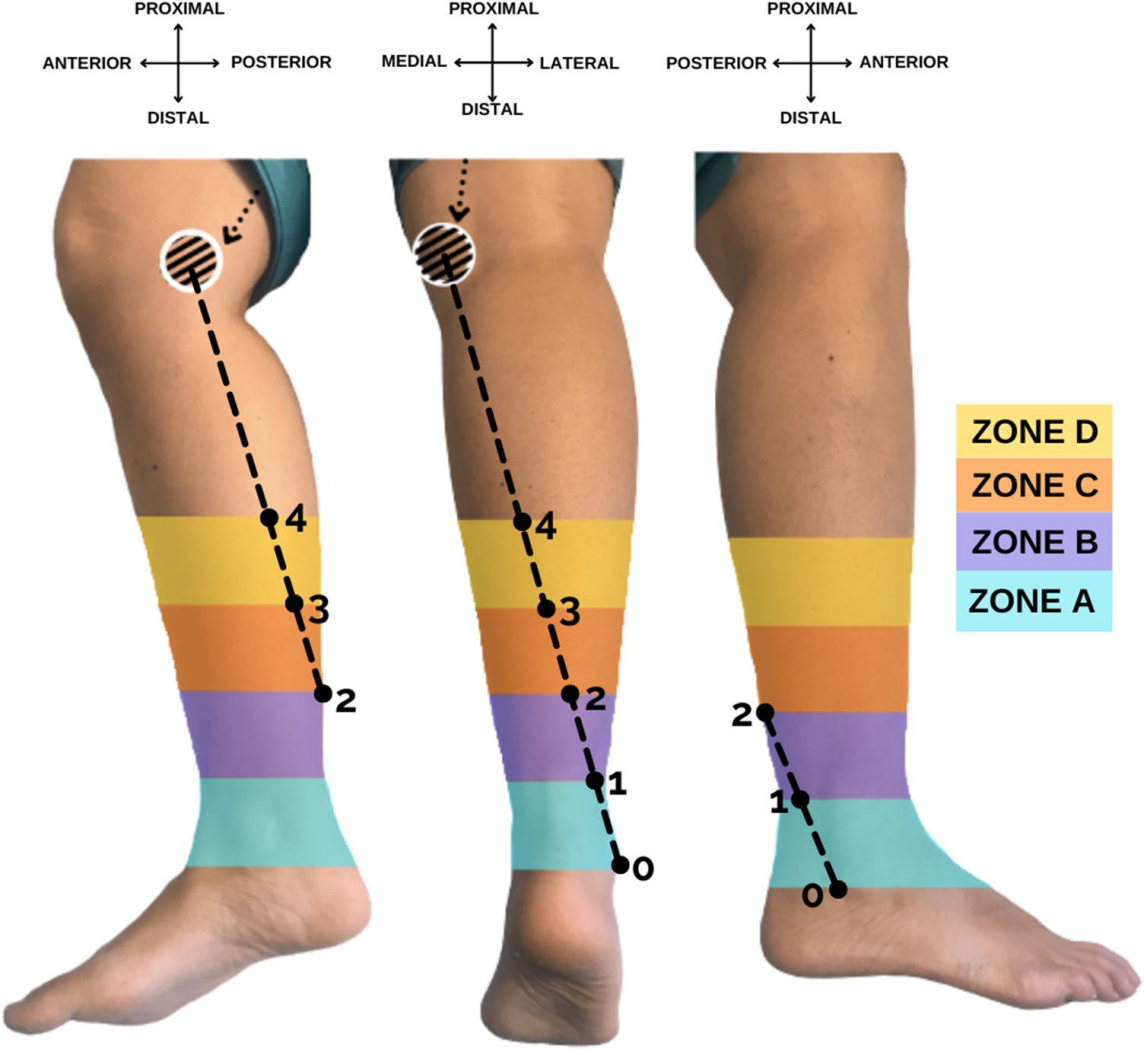
Description of the surface reference line, reference points and zones on a right leg. From left to right, medial, posterior, and lateral views of the leg. Striped circle: medial femoral condyle; Dotted arrow: hamstring tendon; Dotted line: surface reference line; Point 0: fibular tip. Subdivision of the distal half of the leg into five points and four zones listed from distal to proximal

